# Supplementary figures and images for: Significant adhesion reduction and time saving in pediatric heart surgery with 4DryField PH: A retrospective, controlled study
Source: PLoS One. 2022 Nov 17;17(11):e0277530. doi: 10.1371/journal.pone.0277530 (PMC9671326; doi:10.1371/journal.pone.0277530)

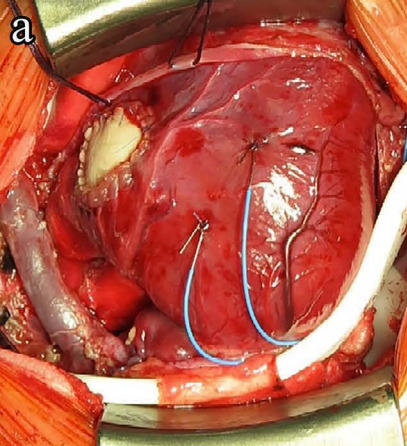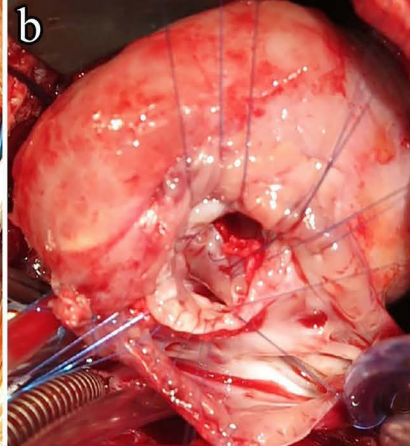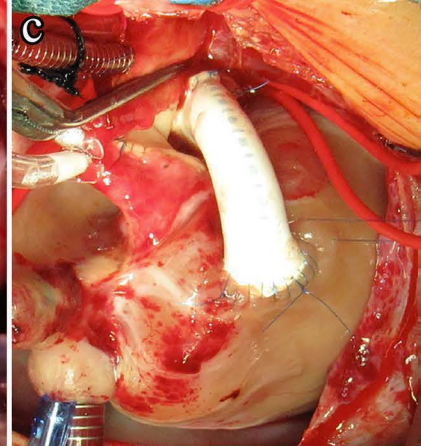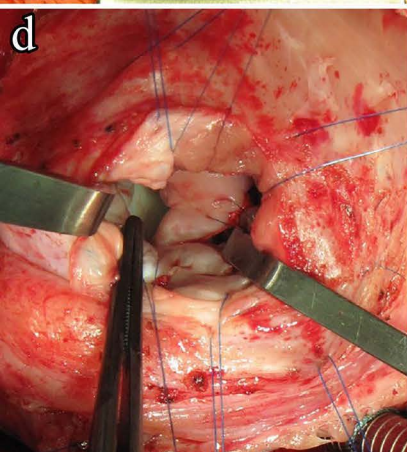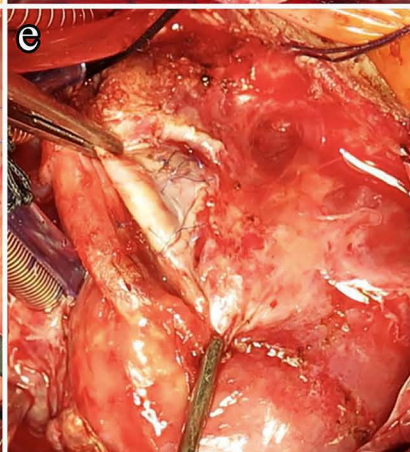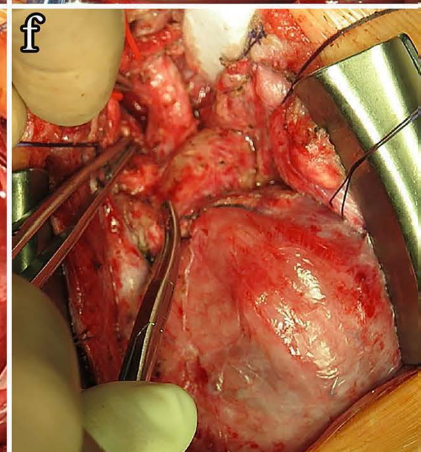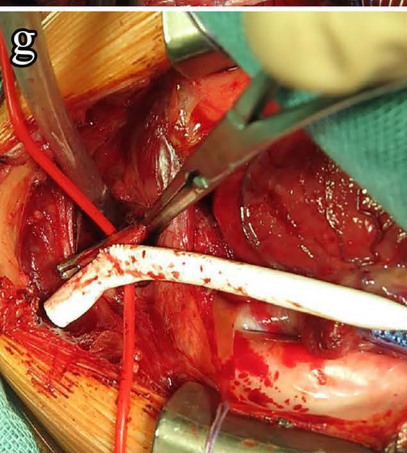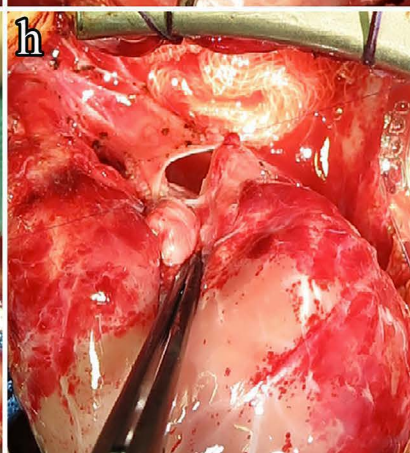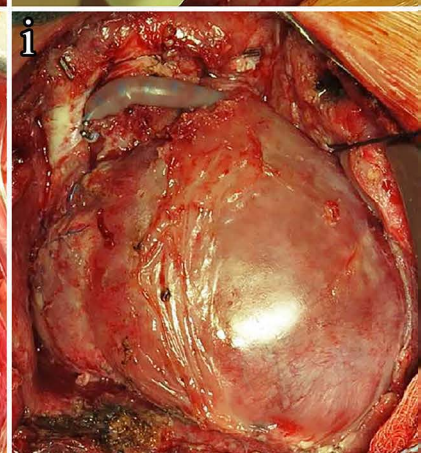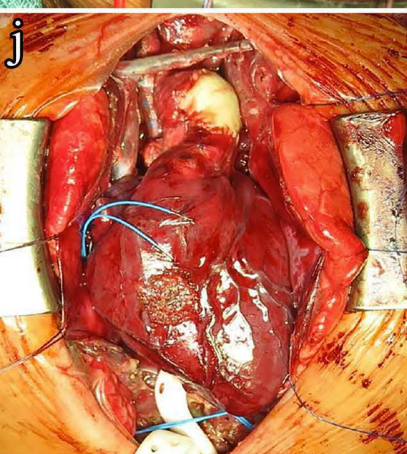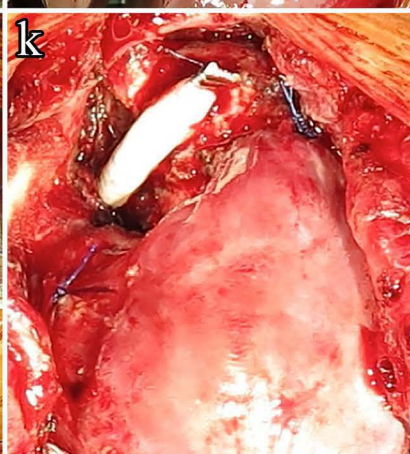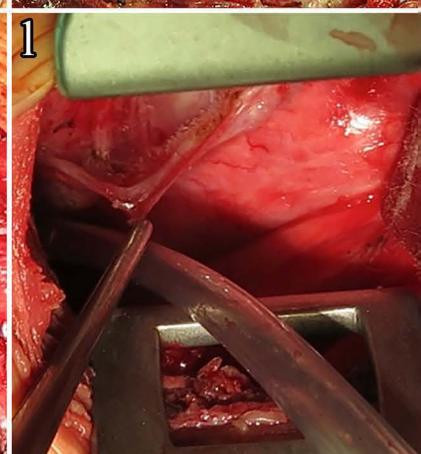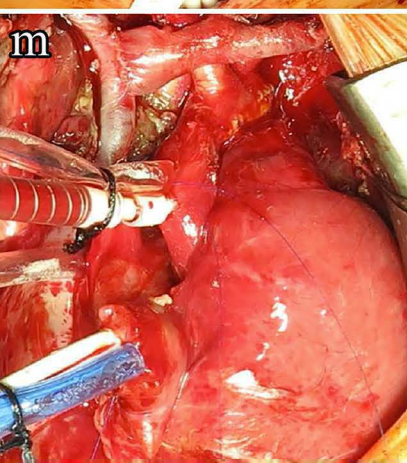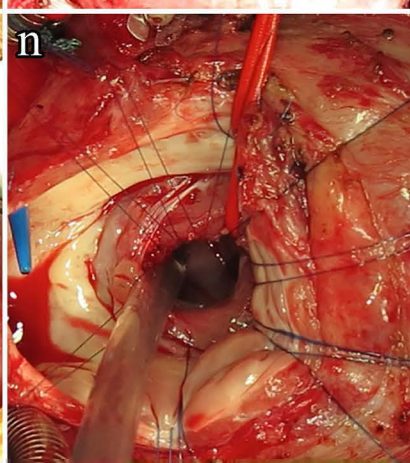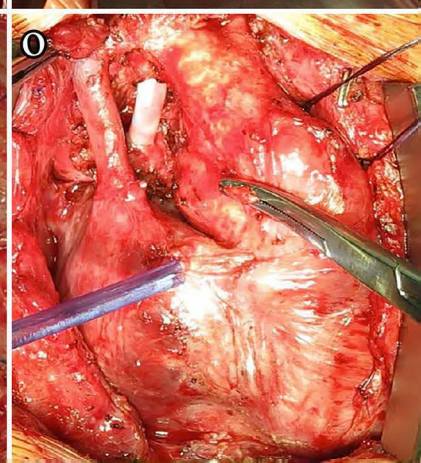

Supplement: S1 Fig — Hover over subfigures for patient and surgery attribution. (PDF) [file pone.0277530.s001.pdf]

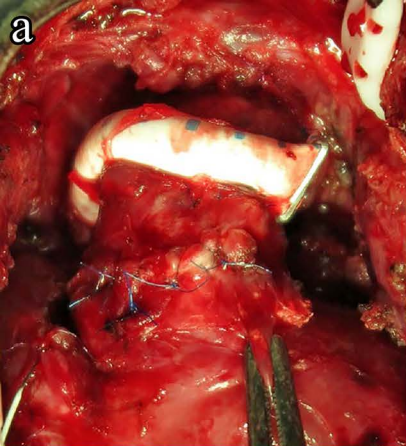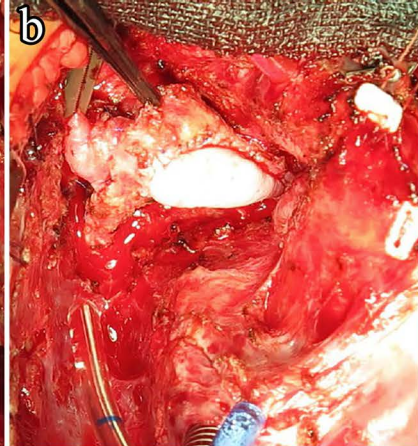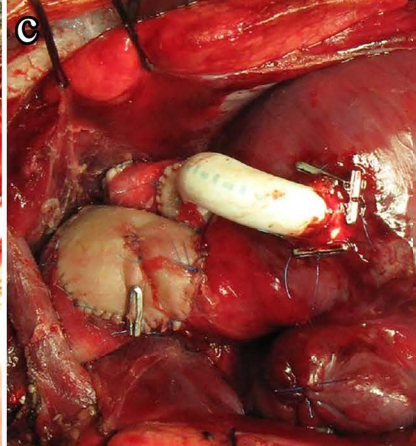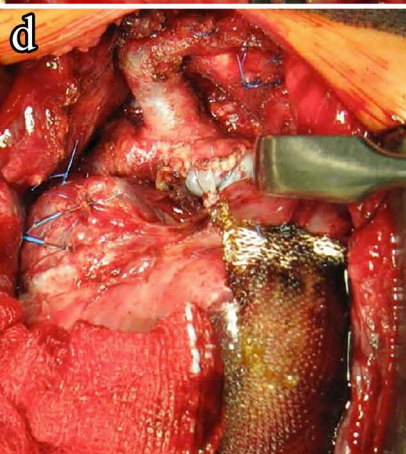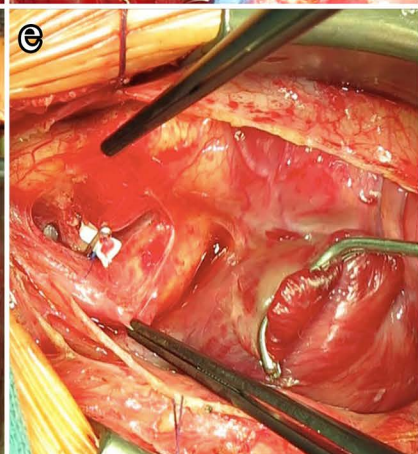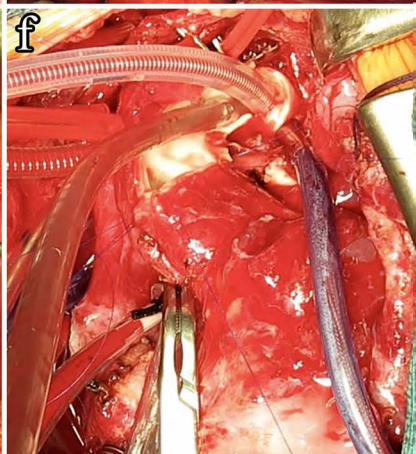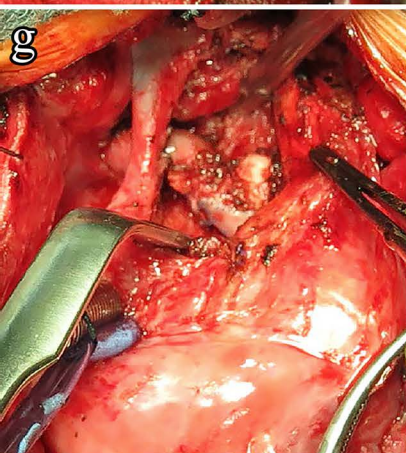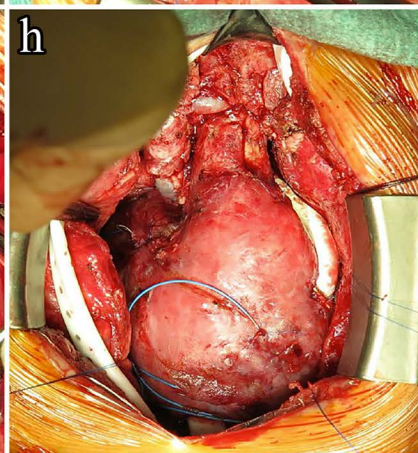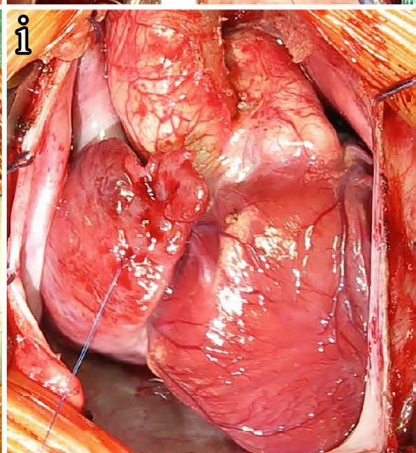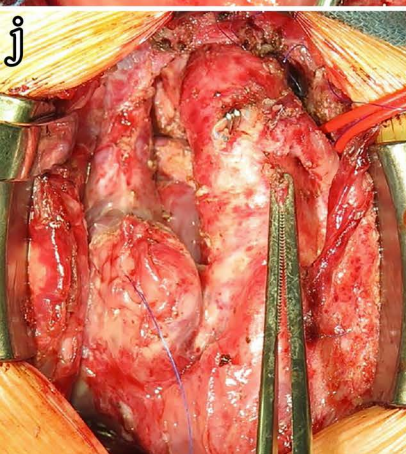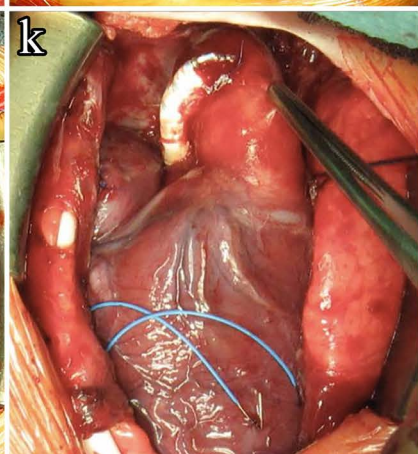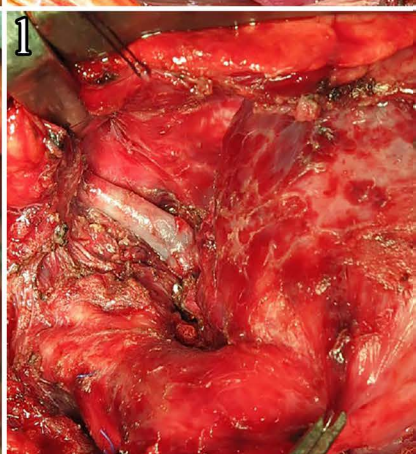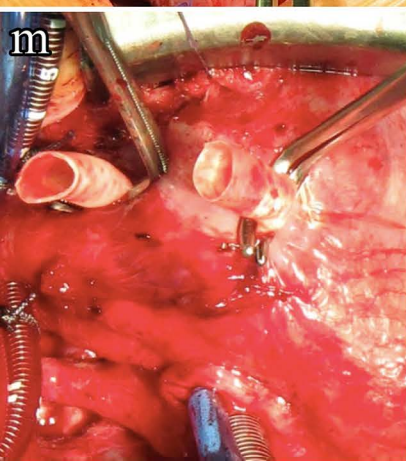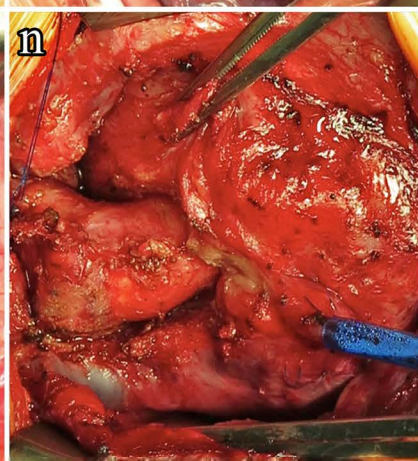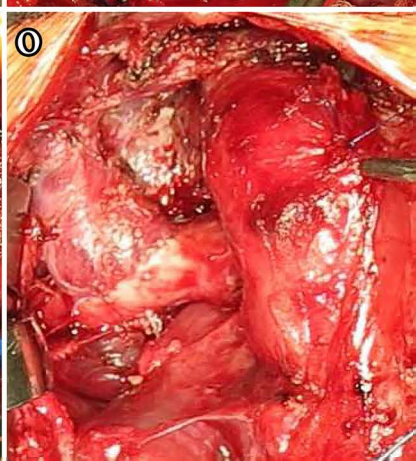

Supplement: S2 Fig — Hover over subfigures for patient and surgery attribution. (PDF) [file pone.0277530.s002.pdf]
