# Supplementary material for: Significant adhesion reduction and time saving in pediatric heart surgery with 4DryField PH: A retrospective, controlled study
Source: PLoS One. 2022 Nov 17;17(11):e0277530. doi: 10.1371/journal.pone.0277530 (PMC9671326; doi:10.1371/journal.pone.0277530)
Supplement: S1 Table — (PDF) [file pone.0277530.s003.pdf]

| Patient ID | Score | Description |
|---|---|---|
| 1 | 1.5 | Pericardium with reflective surface, no hemorrhage, coronary vessels identifiable, dissection without electro coagulation |
| 2 | 1.5 | Pericardium with reflective surface, minor petechial hemorrhages, coronary vessels identifiable, dissection without electro coagulation |
| 3 | 1.5 | Pericardium with reflective surface, no adhesion formation of the ventricles within the pericardial cavity, corona vessels identifiable, large vessels easy to dissect, dissection without electro coagulation |
| 4 | 1.5 | Pericardium with reflective surface, no hemorrhage, coronary vessels identifiable, dissection without electro coagulation |
| 4 (3rd surgery) | 1.5 | Pericardium with reflective surface, no hemorrhage, coronary vessels identifiable, dissection without electro coagulation |
| 5 | 1.5 | Pericardium with reflective surface, slight scar formation around the large vessels, ventricular pericardium slightly thickened without adhesion formation, coronary vessels identifiable, dissection without electro coagulation |
| 5 (3rd surgery) | 1.5 | Pericardium with reflective surface, slight scar formation around the large vessels, ventricular pericardium slightly thickened without adhesion formation, coronary vessels identifiable, dissection without electro coagulation |
| 6 | 2.0 | Pericardium with reflective surface, slight scar formation around the large vessels, minor adhesion formation in the area of ventricles, coronary vessels identifiable, minor electro coagulation necessary |
| 7 | 2.5 | No adhesions of ventricles within the pericardial cavity, corona vessels identifiable, some scar formation around the large vessels, in this area minor electro coagulation necessary |
| A | 2.5 | Dense adhesions to the posterior sternum, right ventricle with minor adhesions, left ventricle completely adhesive to the pericardial cavity, electro coagulation for adhesiolysis necessary, dense scar formation in the area of large vessels |
| B | 2.5 | Scarred adhesions of the thickened pericardium, electro coagulation for adhesiolysis necessary, multiple bleeding sites at the myocardial surface necessitating local application of hemostatic patch, dense scars around the large vessels |
| C | 3.0 | Pericardium thickened, dense adhesions to the posterior sternum, adhesion formation throughout the pericardium, some electro coagulation necessary |
| D | 1.5 | Pericardium with reflective surface, coronary vessels identifiable, no adhesion formation in the area of atria and ventricles, mild scar formation around large vessels, dissection without electro coagulation |
| E | 3.0 | Dense adhesions to the posterior sternum, scarred adhesions throughout the thickened pericardium, electro coagulation necessary, dense scar formation around the large vessels |
| F | 3.0 | Adhesions throughout the pericardium partially scarred, electro coagulation for adhesiolysis necessary, dense adhesions to the posterior sternum |
| G | 2.0 | Dense scar formation around large vessels, adhesions between posterior sternum and right ventricle, minor adhesions in the area of left ventricle, minor electro coagulation necessary |
| H | 3.0 | Dense adhesions to the posterior sternum, scarred adhesions throughout the pericardium, electro coagulation necessary, dense scars around the large vessels, coronary vessels not identifiable |
| I | 2.5 | Dense adhesions to the posterior sternum, right atrium and ventricle with minor adhesions, adhesions of the left ventricle throughout the pericardium, scar formation around the large vessels, some electro coagulation necessary |
