# Supplementary material for: Significant adhesion reduction and time saving in pediatric heart surgery with 4DryField PH: A retrospective, controlled study
Source: PLoS One. 2022 Nov 17;17(11):e0277530. doi: 10.1371/journal.pone.0277530 (PMC9671326; doi:10.1371/journal.pone.0277530)
Supplement: Attachment — Submitted filename: Response to Reviewers_.docx [file pone.0277530.s004.docx]

Dear Editor and Reviewer,

thank you very much for your helpful comments.

Concerning the points raised regarding journal requirements and our corresponding actions:

1. Please ensure that your manuscript meets PLOS ONE's style requirements, including those for file naming.
   *We ensured that our manuscript meets PLOS ONE's style requirements by adjusting the names of the figure files, the way tables are cited, the citation style, the format of Supporting Information Citations and the affiliations of the first and fourth author.*
2. Thank you for stating the following in the Competing Interests section.

*We revised the Competing Interests statement as suggested. You can find the full statement in the cover letter.*

Concerning the points raised by the reviewer under point 5 “Review Comments to the Author” and our corresponding actions:

- Regarding the statistically significant reduction in time from skin incision to CPB, there is a confounding variable as the operator was not blinded. Consider highlighting that or include that in the limitation section.
  *We added a sentence to the discussion section starting at line 86 indicating that the fact that the surgeon was not blinded is a confounding variable concerning the result of a statistically significant reduction in time from skin incision to CPB and, therefore, a limitation.*
- Consider having indicators with the pictures

*We did not add indicators to the pictures. Indicators have been omitted from the illustrations in the publication to keep as many structures visible as possible. Unless absolutely necessary, we would therefore like to keep the illustrations as they are.*

- Small number and single center study should also be included in the limitation section.

*We added a sentence in the discussion section starting at line 71 pointing out the small patient number and single center design as limitations.*

We hope that the manuscript is now acceptable for publication in *PLOS ONE*.

Yours Sincerely

Prof. Dr. med. Joachim Laas
