# Supplementary material for: Significant adhesion reduction and time saving in pediatric heart surgery with 4DryField PH: A retrospective, controlled study
Source: PLoS One. 2022 Nov 17;17(11):e0277530. doi: 10.1371/journal.pone.0277530 (PMC9671326; doi:10.1371/journal.pone.0277530)
Supplement: Attachment — Submitted filename: Response to Reviewers.docx [file pone.0277530.s005.docx]

Dear Editor and Reviewer,

thank you very much for your helpful comments.

Concerning the points raised by the reviewer under point 6 “Review Comments to the Author” and our corresponding actions:

1) I assume no gore membranes were used, even over the rv-pa homografts? These can affect adhesion formation.
   *The assumption is correct. We clarified this by adding a sentence to the Methods and materials chapter stating that no other adhesion prevention measures like the usage of GoreTex membranes were used in any of the patients.*
2) Isn't the definition of score 5 adhesion (electro coagulation) based on surgeon preferences rather than visualization? It may be worth noting how many different surgeons were participating in the study to frame the subjective factor.

*We added a sentence to the Methods and materials section stating that two different surgeons operated the patients included in the study. Furthermore, a statement was added to the discussion section listing this as a further limitation.*

3) Line 119 you should clarify is the adhesive barrier was reapplied after each subsequent surgery.

*A clarification was added stating that patients in the intervention group received adhesion prevention treatment during each surgery.*

4) Line 142 why was the mean surgery duration not different? Seems like a statistical change since the time was 20 minutes shorter, but relative to the overall duration would need larger n to show significance.
   *Although the overall surgery duration was 20 minutes shorter, statistical examination performed as described in the manuscript did not yield a significant difference. This is due to the high variance of surgery length which in turn is due to different kind of procedures being included while the patient number is small. The respective sentence in the discussion section has been supplemented with further explanation as well as the outlook that a larger study should be able to receive statistical significance here as well.*
5) Line 212 grammer can be improved, such as a "shows a" instead of "comes to a"
   *The recommendation has been implemented.*
6) Since the authors have a COI involvement with the product, are they also the ones doing the review and unblinded operations focusing on pre-bypass times? If so this could skew the results. If the authors were not the surgeons on these cases, this should be stated to alleviate any COI concerns.
   *Authors with COI involvement (authors 3 and 4) have neither been involved in adhesion scoring nor in any of the surgeries. A respective declaration has been added to the competing interest statement. Furthermore, the Methods and Materials section now states that adhesion scoring was performed by an independent cardiac surgeon.*

We hope that the manuscript is now acceptable for publication in *PLOS ONE*.

Yours Sincerely

Prof. Dr. med. Joachim Laas
